# Diagnostic Evaluation of the Sysmex XN‐1000V Lymphocyte Fluorescence for Differentiating Canine Nodal Large B‐Cell and T‐Cell Lymphoma

**DOI:** 10.1111/vco.70032

**Published:** 2025-12-23

**Authors:** Javier Martínez‐Caro, Marta Lemos, Beatriz Agulla, Ignacio Amarillo‐Gómez, Josep Pastor

**Affiliations:** ^1^ Servei d'Hematologia Clínica Veterinària, Departament de Medicina i Cirurgia Animals Facultat de Veterinària, Universitat Autònoma de Barcelona Bellaterra Spain; ^2^ Zoetis Virtual Laboratory Zoetis Spain Madrid Spain; ^3^ T‐CITO Barcelona Spain

**Keywords:** dog, flow cytometry, fluorescence, haematology analyser, lymphoproliferative, phenotype, scattergram

## Abstract

Canine lymphoma is a common haematopoietic neoplasm. Immunophenotype is a major prognostic factor and may influence treatment recommendations. This study assessed the diagnostic performance of the Sysmex XN‐1000V white blood cell differential (WDF) scattergram to differentiate canine nodal large B‐cell and T‐cell lymphoma, using the percentage of highly fluorescent cells (%HFC) and visual WDF scattergram evaluation. A retrospective study was conducted on data from cases of cytologically diagnosed canine large cell lymphoma. Cases had concurrent lymph node aspirate cell suspensions in saline that were analysed using the Sysmex XN‐1000V and multiparametric flow cytometry (FC) for lymphoma classification as B or T‐cell. Large B‐cell lymphomas (*n* = 86) showed significantly higher %HFC compared to large T‐cell lymphomas (*n* = 17), with a median (IQR) of 50% (36–84) and 9.7% (3.9–19), respectively. The ROC analysis showed an AUC of 0.93, with an optimal cutoff of < 24.15 %HFC for identifying T‐cell lymphoma, achieving 88.24% sensitivity, 87.21% specificity, 57.69% PPV and 97.40% NPV. The following data is expressed as ‘overall‐percentage‐agreement (kappa value)’. Using the previous cutoff, the agreement between the %HFC classification and FC was 88.24% (*κ* = 0.76). Regarding the WDF scattergram evaluation, the intra‐ and inter‐observer agreement were 86.27% (*κ* = 0.71) and 67.65% (*κ* = 0.55), respectively. Agreement between the WDF scattergram evaluation and FC was 77.45% (*κ* = 0.55), and improved to 90.63% (*κ* = 0.74) when just the confident cases were used. In conclusion, a preliminary assessment of the phenotype of canine nodal large cell lymphoma can be made using either the visual inspection of the WDF scattergram or the %HFC. This could serve as a cost‐effective, fast screening tool while awaiting definitive flow cytometry results.

## 1 Introduction

Lymphoma is one of the most common neoplasms affecting dogs, with an estimated incidence rate of 20–100 cases per 100 000 dogs [1]. Multicentric (nodal) lymphoma accounts for the majority of cases by location, representing approximately 75% of all cases [2].

Immunophenotyping refers to the identification of antigens specific to a given lymphocyte lineage, using antibody‐based detection methods. In veterinary medicine, several techniques are currently available, including flow cytometry (FC), immunohistochemistry, immunocytochemistry and immunofluorescence [3]. Traditionally, the importance of immunophenotype relied on the distinction of B‐cell and T‐cell lymphoma, which is considered a major prognostic factor [3] and may influence treatment recommendations [2]. Nowadays, more complete antibody panels enable complete lymphoma subtype classification in many cases, and other molecules with prognostic implications are also assessed [3].

The white blood cell differential (WDF) channel of the Sysmex XN‐1000V haematology analyser (Sysmex Corporation, Norderstedt, Germany) provides information on highly fluorescent cells (HFC), reflecting differences in cellular characteristics. The high fluorescence in the WDF scattergram indicates a high affinity by the cells to the Fluorocell WDF reagent (Sysmex Corporation, Norderstedt, Germany), which stains nucleic acids present in the cytoplasm (mainly RNA) [4]. In human medicine, this fluorescence pattern has been associated with antibody‐synthesising cells (atypical lymphocytes, reactive lymphocytes and plasma cells) [5, 6, 7].

Our aim was to assess the diagnostic performance of the WDF channel from the Sysmex XN‐1000V to differentiate canine nodal large B‐cell and T‐cell lymphoma using the percentage of HFC (%HFC). Secondary objectives included evaluating the utility of visual assessment of WDF scattergrams and cytomorphology for assigning a presumptive phenotype. This new method could provide an affordable and widely accessible way to preliminarily distinguish the phenotype of nodal large cell lymphoma in dogs.

## 2 Materials and Methods

### 2.1 Automated Haematology, Flow Cytometry Analysis and Case Selection

Cell suspensions of canine lymph node aspirates were submitted to our laboratory for diagnostic FC analysis. The samples were stored at 6°C and analysed within 12–48 h after collection. Prior to the FC analysis, the samples were run on the Sysmex XN‐1000V in ‘whole blood’ mode to determine the total nucleated cell count. The multiparametric FC analysis was performed on a CytoFLEX LX Flow Cytometer (Beckman Coulter Inc., Brea, CA, USA) using conventional protocols previously described [8, 9] with canine‐specific and cross‐reactive monoclonal antibodies against canine clusters of differentiation (CD)45, CD18, CD21, CD5, CD3, CD4, CD8, CD25, CD14, CD34, major histocompatibility complex class II and Ki67 (Table S1). The FC data interpretation was conducted using CytExpert Software (Beckman Coulter Inc., Brea, CA, USA). Different leukocyte populations were established based on light scatter and immunophenotype properties (backgating) of viable single events using the viability dye 7‐aminoactinomycin D (SouthernBiotech, Birmingham, AL, USA).

The FC data was interpreted in accordance with the current veterinary bibliography [10, 11]. In summary, a B‐cell lymphoma was diagnosed by the expression of the B‐cell marker CD21 and the absence of the pan‐T‐cell markers (CD3 and CD5). On the contrary, a T‐cell lymphoma was diagnosed by the expression of CD3 and/or CD5, and the absence of CD21. In the present study, this ‘flow cytometry’ phenotype was used as the gold standard for comparison purposes.

During an 18‐month period, all the cases that met the following inclusion criteria were retrospectively included in the study: (i) canine lymph node samples, (ii) cytological diagnosis of lymphoma of ‘intermediate to large’ or ‘large’ cells, (iii) concurrent cytology smear and cell suspension with more than 2000 nucleated cells per microliter and (iv) concurrent analyses on the Sysmex XN‐1000V and multiparametric FC.

### 2.2 Visual WDF Scattergram and Cytomorphology Evaluations

**FIGURE 1:**
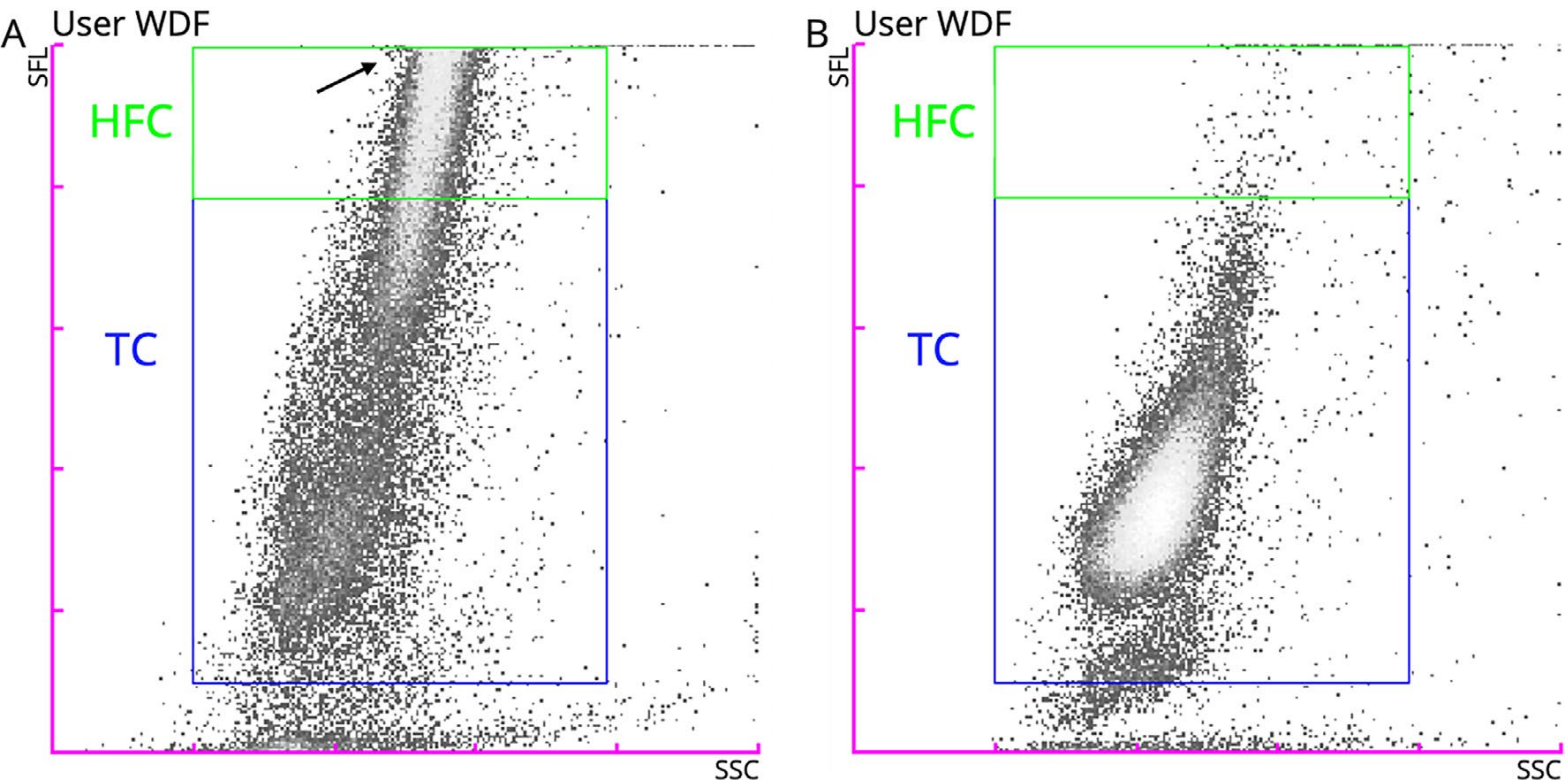
New WDF gate in the manual analysis (extended) from the Sysmex XN‐1000V. (A) Large B‐cell lymphoma aspirate with numerous HFC and part of the main cell population outside the scattergram representation area (arrow). (B) Large T‐cell lymphoma aspirate with most of the cells below the HFC gate and exhibiting low fluorescence intensity. HFC, highly fluorescent cells; SFL, side fluorescence light; SSC, side scatter; TC, total cells; WDF, white blood cell differential.

For these evaluations, we used all the T‐cell lymphoma cases and an equal number of B‐cell lymphoma cases, selected in reverse chronological order from the end of the study period. Three observers (I.A.‐G., J.P. and B.A.) blindly and independently evaluated the WDF scattergrams from the Sysmex XN‐1000V. The scattergrams were shared in their raw form (without gating or numerical information) and in a grey density gradient. For each case, the observers answered the following questions: ‘How would you classify this lymphoma?’ and ‘How confident are you for this classification?’, with the following possible answers: ‘B‐cell versus T‐cell lymphoma’ and ‘confident versus non‐confident’, respectively. Before answering the questions, Figure 1 was shown to the observers. They were given the following information: the study's inclusion criteria (see above) and the expected typical fluorescence features for each entity: (i) B‐cell lymphoma cases: higher concentration of HFC and usually several events above the side fluorescence light reading area and (ii) T‐cell lymphoma cases: lower concentrations of HFC. The cases were randomly ordered and shared with the observers, who had to answer both questions in all the cases. A second round of evaluation, with a different random order of cases and conducted 1 week later, was used for intra‐observer agreement calculations. The answers from the first round were used to calculate the inter‐observer agreement and the agreement between the visual WDF assessment and the FC. For the latter, the analysis was repeated after excluding the ‘non‐confident cases’.

Additionally, the cytological smears of the same lymphoma cases were digitalized using the MoticEasyScan One (MoticEurope SLU, Barcelona, Spain). The whole slide images were devoid of any original names or identification numbers and were placed in a random order. The files were shared with the three observers, who evaluated them blindly and were asked ‘How would you classify this lymphoma?’ using the current veterinary bibliography [12, 13, 14]. The possible answers were ‘B‐cell versus T‐cell lymphoma’. The agreement between the cytomorphological phenotype and the FC was calculated.

### 2.3 Development and Evaluation of a New WDF Gate

A new gate on the WDF channel was created using the manual analysis (extended) in software version 3 (3.07–00, Sysmex) to quantify the %HFC, as seen in Figure 1. This gate was saved as a ‘new species’ to allow its use in future cases and to retrospectively apply it to previously analysed cases, including all those included in the present study. The gate information for the WDF channel in the manual analysis (extended) is shown in Table S2.

The intra‐assay imprecision of the new gate was determined at three levels (low, medium and high %HFC), analysing 10 replicates of leftover samples. To assess its acceptability, the total allowable error (TEa) recommendations from the ASVCP guidelines were used [15]. Although no specific recommendations for this new variable were available, the 15% TEa for neutrophils and lymphocytes was used for the medium and high %HFC levels, and the 50% TEa for eosinophils was used for the low %HFC level [15]. The calculated intra‐assay imprecision was considered satisfactory if it was < 0.25 × TEa [16].

The %HFC was compared between B‐cell and T‐cell lymphoma cases. A receiver operating characteristic (ROC) analysis was performed. After determining a cutoff for phenotype differentiation, the agreement between the resulting phenotype and the FC was assessed.

### 2.4 Statistical Analysis

Data collection was performed on Microsoft Excel software (16.78.3 version). The kappa analyses were carried out on the free website: https://rbiostatistics.com/ (accessed on 2 January 2025). Cohen's kappa was used for all the agreement analyses except for the inter‐observer agreement of the visual WDF evaluation, for which the Fleiss kappa was used. Kappa values were interpreted as follows: < 0, no agreement; 0.00–0.20, slight agreement; 0.21–0.40, fair agreement; 0.41–0.60, moderate agreement; 0.61–0.80, substantial agreement; 0.81–1.00, almost perfect agreement [17]. Additionally, the overall percentage agreement was also provided for all the agreement calculations (number of agreements ÷ total number of items × 100). The GraphPad Prism 9 software was used for all the other statistical analyses. A *p*‐value of less than 0.05 was considered statistically significant. The normal distribution of the data was assessed using the Shapiro–Wilk test, in order to select subsequent parametric or non‐parametric tests. The %HFC was compared between both groups using the Mann–Whitney test. The diagnostic performance of the %HFC for differentiating T‐cell versus B‐cell lymphoma cases was evaluated using ROC analysis. Due to their higher prevalence, the B‐cell lymphoma cases were assigned as the ‘control group’ category for ROC calculations. The 95% confidence intervals (CI) were calculated using the Wilson/Brown method. An optimal cutoff was derived from the ROC analysis using the Youden index. The positive predictive value (PPV) and negative predictive value (NPV) were calculated for each threshold using the true positive, true negative, false positive and false negative values, as previously described [18].

## 3 Results

A total of 103 lymphoma cases met the inclusion criteria and were included in the study. Using FC, 17 cases were classified as large T‐cell lymphoma and 86 cases as large B‐cell lymphoma.

### 3.1 Visual WDF Scattergram and Cytomorphology Evaluations

The raw WDF scattergram and the cytology smear of 34 lymphoma cases were blindly evaluated by the three observers. The intra‐observer and inter‐observer agreement for the phenotype derived from the visual WDF scattergram evaluation was substantial and moderate, respectively (Table 1). The overall agreement between the phenotype derived from the visual WDF scattergram evaluation and the FC was 77.45% (0.55 kappa value) (Table 1). Observers 1, 2 and 3 felt ‘confident’ in the visual WDF scattergram evaluation in 16, 24 and 28 cases, respectively. When just those cases were used for analysis, the overall agreement improved significantly and was 90.63% (0.74 kappa value) (Table 1). The overall agreement of the cytomorphological phenotype with the phenotype established by FC was 78.43% (0.57 kappa value) (Table 1).

**TABLE 1:** Agreement metrics using overall percentage agreement and Cohen's or Fleiss' kappa values. The intra‐ and inter‐observer agreement for the visual WDF scattergram evaluation is provided. Comparisons between the lymphoma phenotype obtained using (i) the visual WDF method (all cases and only ‘confident’ cases), cytomorphology or %HFC and (ii) flow cytometry are provided.

	Overall % agreement: mean (min.–max.)	Kappa: mean (min.–max.)
Visual WDF scattergram evaluation		
Intra‐observer agreement	86.27 (79.41–91.18)	0.71 (0.58–0.80)
Inter‐observer agreement	67.65^a^	0.55^b^
Visual WDF vs. FC phenotype	77.45 (73.53–82.35)	0.55 (0.47–0.65)
Visual WDF vs. FC phenotype (only ‘confident’ cases)	90.63 (82.14–93.75)	0.74 (0.59–0.88)
Cytomorphological vs. FC phenotype	78.43 (76.47–82.35)	0.57 (0.53–0.65)
%HFC vs. FC phenotype^c^	88.24	0.76

*Note*: Moderate and substantial kappa agreement are indicated by orange and green, respectively.

Abbreviations: %HFC, percentage of highly fluorescent cells; FC, flow cytometry; max., maximum; min., minimum; vs., versus; WDF, white blood cell differential.

^a^ Cases in which the three observers agreed.

^b^ Fleiss kappa analysis.

^c^ The lymphoma phenotype assigned using a cutoff of < 24.15% HFC is used for the comparison with the gold standard.

### 3.2 New WDF Gate Evaluation

**FIGURE 2:**
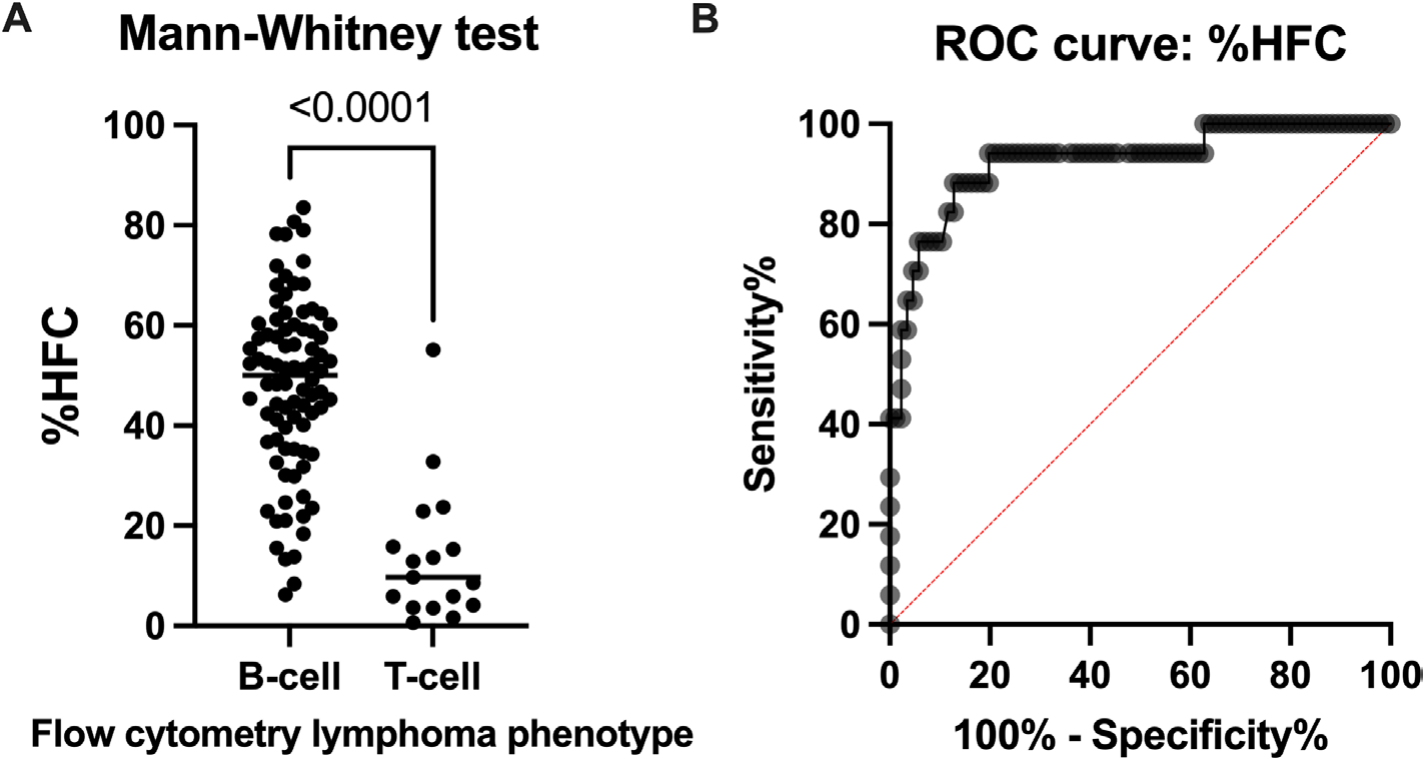
Differentiation of canine large B‐cell and T‐cell lymphoma using %HFC calculated on the WDF channel from the Sysmex XN‐1000V. (A) Scatter plot showing the different %HFC in both lymphoma phenotypes. (B) ROC curve for %HFC in distinguishing B‐cell from T‐cell lymphoma in dogs. %HFC, percentage of highly fluorescent cells; ROC, receiver operating characteristic.

The three cases used for the intra‐assay imprecision study of the new WDF gate had a mean of 1.9, 26 and 57 %HFC. The coefficients of variation were 12%, 3% and 1%, for the low, medium and high %HFC, respectively. The %HFC was significantly different between B‐cell and T‐cell lymphoma cases (Figure 2A), with a median and interquartile range (IQR) of 50 (36–84) and 9.7 (3.9–19), respectively. The ROC analysis showed an Area Under the Curve (AUC) of 0.93 (95% CI 0.85–1). The ROC curve is represented in Figure 2B. The optimal decision limit was < 24.15% HFC, selected using the Youden index, which corresponds with the most upper left point on the ROC curve [19]. This cutoff showed a sensitivity (Se) of 88.24% (95% CI 65.66%–97.91%), a specificity (Sp) of 87.21% (95% CI 78.53%–92.71%), PPV of 57.69%, NPV of 97.40% and a likelihood ratio (LR) of 6.9 for identifying T‐cell lymphoma. The agreement between the phenotype derived from the < 24.15% HFC cutoff and the FC can be found in Table 1. Alternative cutoffs for identifying large T‐cell lymphoma could be < 15.40% HFC (Se 70.59% [95% CI 46.87%–86.72%], Sp 95.35% [95% CI 88.64%–98.18%], PPV of 75.00%, NPV of 94.25% and LR 15.18) or < 33.55% HFC (Se 94.12% [95% CI 73.02%–99.70%], Sp 80.23% [95% CI 70.60%–87.28%], PPV of 48.48%, NPV of 98.57% and LR 4.7).

## 4 Discussion

Immunophenotyping is part of the diagnostic protocol for canine lymphoma, as it provides information that is considered a major prognostic factor [3]. Additionally, in some institutions different chemotherapeutic approaches could be considered depending on the specific phenotype [3]. Although there is no doubt that immunophenotyping is always recommended, in many cases this is not performed, mainly due to economic concerns.

We evaluated, for the first time, the diagnostic performance of the WDF scattergram from the Sysmex XN‐1000V to differentiate large B‐cell and T‐cell lymphoma in dogs. The predominance of B‐cell lymphoma in our population is in agreement with the B‐cell lymphoma prevalence described in the bibliography [20, 21, 22]. Our results support that either the %HFC and the visual WDF scattergram evaluation could be used to differentiate canine nodal large B‐cell and T‐cell lymphoma.

The substantial and moderate agreement for the intra‐ and inter‐observer agreement in the visual WDF scattergram evaluation was judged to be satisfactory. The agreement between the visual WDF scattergram evaluation and the FC phenotype improved from moderate to substantial when only the cases for which the observers felt confident were evaluated. The agreement between the phenotypes established by cytomorphology and FC was similar to the agreement between the visual WDF scattergram evaluation. Martini et al. [22] described lower agreement between cytology and histopathology with immunohistochemistry; however, their results are not comparable to ours because the authors considered other diagnostic categories, not just B‐cell versus T‐cell lymphoma differentiation.

The intra‐assay imprecision of the new gate was satisfactory and the %HFC values differed significantly between the two lymphoma populations, enabling calculation of different cutoffs and their associated diagnostic performance. The substantial agreement between the lymphoma phenotype determined with the optimal %HFC cutoff and FC suggests that this method could be a practical adjunct in routine diagnostics.

The typical WDF scattergram characteristics of large T‐cell lymphoma (low numbers of HFC) were similar to those observed in other conditions such as T‐zone lymphoma or hyperplastic/reactive lymph nodes (Figure S1). This is the reason for the strict inclusion criteria of our validation cohort and why this WDF scattergram information should only be interpreted to differentiate large B‐cell versus large T‐cell lymphoma cases in already confirmed lymphoma samples (either cytologically or by other means). Furthermore, the WDF scattergram characteristics in other lymphoma subtypes (i.e., diffuse small B‐cell lymphoma) have not been investigated to date. Although there is discrepancy regarding the appropriateness of providing a cytological grade for lymphoma, Raskin proposed two grades based on the updated Kiel classification scheme, depending on cell size and mitotic count [12]. Low‐grade lymphoma should have low mitotic count and small cell size, while high‐grade lymphoma should have moderate or high mitotic count and medium or large cell size. This classification depends on the skills of the people reviewing the cytology and how familiar they are with these criteria. Regardless of the usefulness of cytology for grading lymphoma, it is clear that most of the low‐grade lymphomas, including the T‐zone lymphoma subtype, are characterised by a small cell size. The inclusion criterion of a cytological diagnosis of ‘intermediate to large’ or ‘large’ cell lymphoma should be sufficient to exclude virtually all cases of low‐grade lymphoma.

There are several possible explanations for the increased fluorescence of the neoplastic B‐cells in the WDF scattergram. The most plausible explanation could be a higher RNA concentration, since one of the main functions of B‐cells is immunoglobulin production. Previous studies demonstrated that non‐T lymphocytes (mainly B lymphocytes) had higher RNA content per cell compared to some subsets of T lymphocytes [23]. The high fluorescence in the WDF scattergram indicates a high affinity by the cells to the Fluorocell WDF reagent (Sysmex Corporation, Norderstedt, Germany), which contains polymethine dye and stains nucleic acids present in the cytoplasm (mainly RNA) [4]. Some studies have evaluated the role of different Sysmex automated haematology analysers in the enumeration of high fluorescent lymphocytes in human blood, which corresponded to antibody‐synthesising cells (atypical or reactive lymphocytes and plasma cells) [5, 6, 7]. We also noted that suspensions of plasma cells from cutaneous plasma cell tumours showed very high fluorescence on the WDF scattergram (authors' observation). In general, the fluorescent pattern of B‐cell lymphoma cases usually correlates with the deep basophilic staining of their cytoplasm observed in cytology using Romanowsky stains.

Another possibility is that cell autofluorescence could have contributed to the fluorescence recorded from the 633 nm laser in the haematology analyser. This emission pattern (excitation from the 633 nm laser, detected in the 650–670 nm range) has been reported by other researchers [24] and encountered infrequently by these authors. However, due to the high brightness of the polymethine dye and the lack of higher autofluorescence on the CytoFLEX LX Flow Cytometer (660 nm detector) in cases with the highest WDF fluorescence compared to cases with the lowest WDF fluorescence (data not shown), we hypothesise that this potential phenomenon has a minor influence. To our knowledge, WDF scattergrams cannot be generated in the Sysmex XN‐1000V without using the WDF reagent, which would be helpful to assess any possible autofluorescence interference in the samples. Some endogenous fluorophores have been described in human WBC, including flavin adenine dinucleotide (FAD), reduced nicotinamide adenine dinucleotide (NADH) and reduced nicotinamide adenine dinucleotide phosphate (NADPH) [25]. These are unlikely to contribute to WDF fluorescence because their excitation and emission ranges differ from those used by the Sysmex XN analyser.

This new method for discriminating B‐cell and T‐cell lymphoma cases could be useful in different scenarios. In some clinical settings, it is not possible to perform all recommended diagnostic tests due to limited availability of technology or financial constraints. The analyser used in this study does not require specialised technical personnel, is widely available in reference laboratories and large veterinary hospitals, and costs less per test than FC or other immunophenotyping methodologies. Although this new approach does not substitute the use of FC or other traditional immunophenotyping techniques, it is likely that more animals could undergo this investigation, particularly in areas with lower socioeconomic profiles. Other possible applications in laboratories performing FC include refining the antibody panel based on the preliminary phenotype, providing the preliminary phenotype to clinicians when samples are cryopreserved and analysed in batches on specific days, or reporting a provisional phenotype when cases lack sufficient cellularity for multiparametric FC.

The main limitations of this work are those inherent to the retrospective study design, primarily the reliance on existing data and cases. We also lacked clinical information on some cases, including whether the animals were receiving any treatment (chemotherapy or glucocorticosteroids), which could have had some effect on our results. Additionally, the new WDF gate alone could not differentiate between large T‐cell lymphoma and other conditions such as non‐neoplastic lymph nodes or T‐zone lymphoma, as discussed above.

In conclusion, either the visual inspection of the WDF scattergram of the Sysmex XN‐1000V or the %HFC derived from the same scattergram could be used for a preliminary approach to the phenotype in canine nodal large cell lymphoma. Although this new methodology does not substitute the conventional immunophenotypic methodologies such as FC, it could be useful in some clinical settings.

## Funding

The authors have nothing to report.

## Ethics Statement

In compliance with local legislation, ethical approval was not required for this study due to its retrospective nature. Samples were collected and analysed as part of the routine diagnostic investigation protocol. All owners provided written informed consent.

## Conflicts of Interest

The authors declare no conflicts of interest.

## Supporting information


**Figure S1:** Canine lymph node aspirate analysis using the new WDF gate using the manual analysis (extended) mode from the Sysmex XN‐1000V. (A) Case of a reactive lymph node diagnosed on flow cytometry, showing 8.1% HFC. (B) Case of a T‐zone lymphoma diagnosed on flow cytometry, showing 1.8% HFC. In both cases, there are few events in the HFC region.

**Data S1:** vco70032‐sup‐0002‐TableS1‐S2.docx.

## Data Availability

The data that support the findings of this study are available from the corresponding author upon reasonable request.
