# Supplementary figures and images for: Diagnostic Evaluation of the Sysmex XN‐1000V Lymphocyte Fluorescence for Differentiating Canine Nodal Large B‐Cell and T‐Cell Lymphoma
Source: Vet Comp Oncol. 2025 Dec 23;24(1):121–7. doi: 10.1111/vco.70032 (PMC12875747; doi:10.1111/vco.70032)

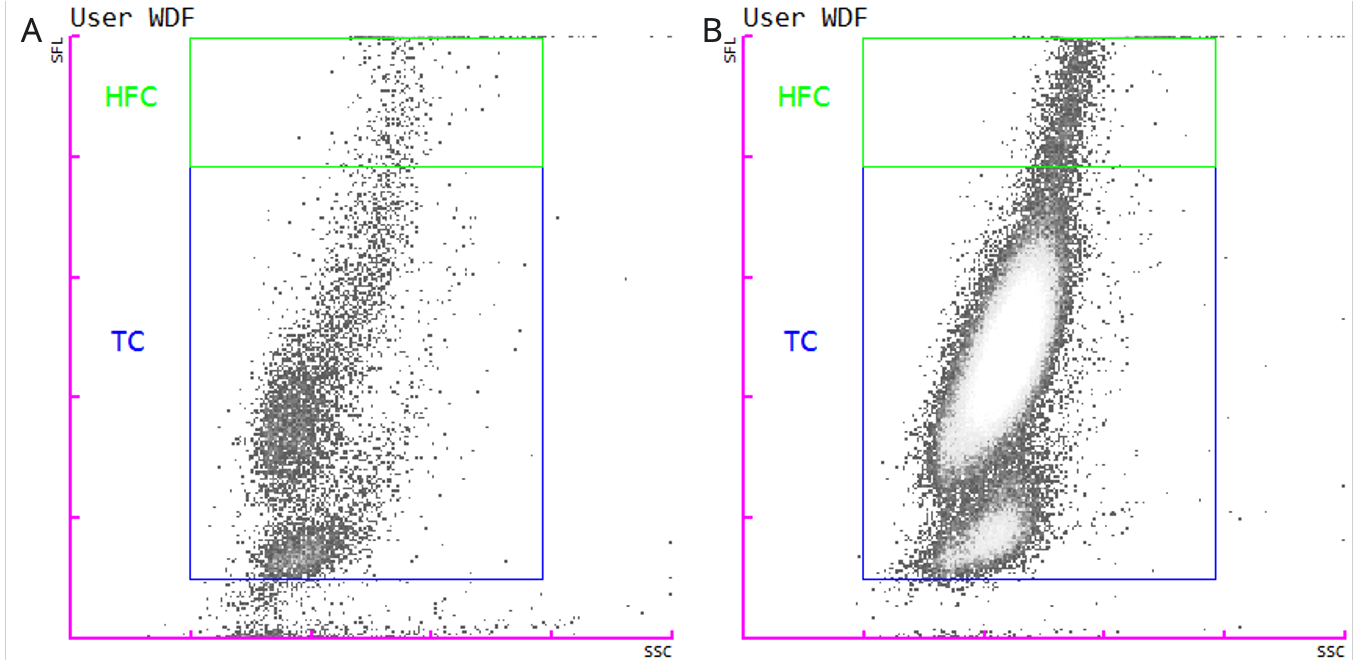

Supplement: Supplementary file 1 — Figure S1: Canine lymph node aspirate analysis using the new WDF gate using the manual analysis (extended) mode from the Sysmex XN‐1000V. (A) Case of a reactive lymph node diagnosed on flow cytometry, showing 8.1% HFC. (B) Case of a T‐zone lymphoma diagnosed on flow cytometry, showing 1.8% HFC. In both cases, there are few events in the HFC region. [file VCO-24-121-s001.tiff]
