# Supplementary material for: Diagnostic Evaluation of the Sysmex XN‐1000V Lymphocyte Fluorescence for Differentiating Canine Nodal Large B‐Cell and T‐Cell Lymphoma
Source: Vet Comp Oncol. 2025 Dec 23;24(1):121–7. doi: 10.1111/vco.70032 (PMC12875747; doi:10.1111/vco.70032)
Supplement: Supplementary file 2 — Data S1: vco70032‐sup‐0002‐TableS1‐S2.docx. [file VCO-24-121-s002.docx]

Supplementary Table 1

| **Antigen** | **Conjugation** | **Clone** | **Specificity** | **Isotype** | **Manufacturer** |
| --- | --- | --- | --- | --- | --- |
| CD45 | Alexa Fluor 647 | YKIX716.13 | Rat anti-canine | IgG2b | Bio-Rad, Oxford, UK |
| CD18 | APC | CA1.4E9 | Mouse anti-canine | IgG1 | Bio-Rad, Oxford, UK |
| CD21 | PE | CA2.1D6 | Mouse anti-canine | IgG1 | Bio-Rad, Oxford, UK |
| CD5 | FITC | YKIX322.3 | Mouse anti-canine | IgG2a | Bio-Rad, Oxford, UK |
| CD5 | Pacific Blue | YKIX322.3 | Mouse anti-canine | IgG2a | Bio-Rad, Oxford, UK |
| CD3 | FITC | CA17.2A12 | Mouse anti-canine | IgG1 | Bio-Rad, Oxford, UK |
| CD4 | Pacific Blue | YKIX302.9 | Rat anti-canine | IgG2a | Bio-Rad, Oxford, UK |
| CD8 | Alexa Fluor 700 | YCATE55.9 | Rat anti-canine | IgG1 | Bio-Rad, Oxford, UK |
| CD25 | Super Bright 436 | P4A10 | Mouse anti-canine | IgG1 | Thermo Fisher Scientific, Waltham, MA, USA |
| CD14 | PE | TÜK4 | Mouse anti-human | IgG2a | Bio-Rad, Oxford, UK |
| CD34 | Alexa Fluor 647 | 1H6 | Mouse anti-canine | IgG1 | Bio-Rad, Oxford, UK |
| MHCII | FITC | YKIX334.2 | Rat anti-canine | IgG2a | Bio-Rad, Oxford, UK |
| Ki67 | FITC | MIB-1 | Mouse anti-human | IgG1 | Dako Omnis, Santa Clara, CA, USA |

List of canine-specific and cross-reactive monoclonal antibodies used in the flow cytometric evaluation. Notes: APC, allophycocyanin; CD, cluster of differentiation; FITC, fluorescein isothiocyanate; MHCII, major histocompatibility complex class II; PE, phycoerythrin.

Supplementary Table 2

Gate information of the WDF channel in the manual analysis (extended).

| **Total cells** | | **High fluorescent events** | |
| --- | --- | --- | --- |
| **X** | **Y** | **X** | **Y** |
| 50 | 25 | 50 | 200 |
| 200 | 25 | 200 | 200 |
| 200 | 255 | 200 | 255 |
| 50 | 255 | 50 | 255 |
